# Supplementary material for: Miniature optical planar camera based on a wide-angle metasurface doublet corrected for monochromatic aberrations
Source: Nat Commun. 2016 Nov 28;7:13682. doi: 10.1038/ncomms13682 (PMC5133709; doi:10.1038/ncomms13682)
Supplement: Supplementary Information — Supplementary Figures 1-7, Supplementary Tables 1-2, Supplementary Note 1 and Supplementary References [file ncomms13682-s1.pdf]

## SUPPLEMENTARY FIGURES

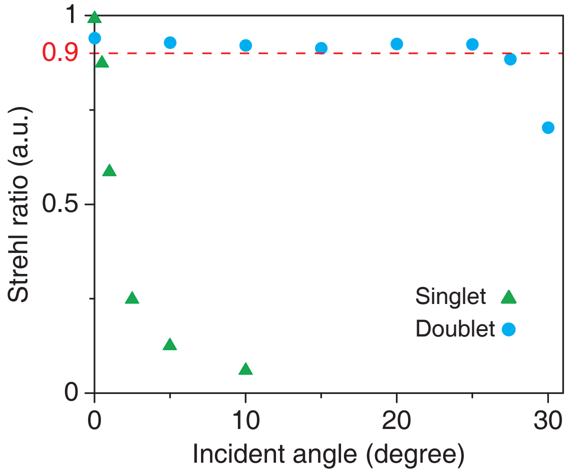


**Supplementary Figure 1**. **Strehl ratio of the singlet and doublet metasurface lenses**. Strehl ratio is the ratio of the volume under the 2D MTF of a lens to the volume under the 2D MTF of a diffraction limited lens with the same NA. The red dashed line shows the Strehl ratio value of 0.9 which we have used as a threshold for referring to the focal spot as being "nearly diffraction limited".

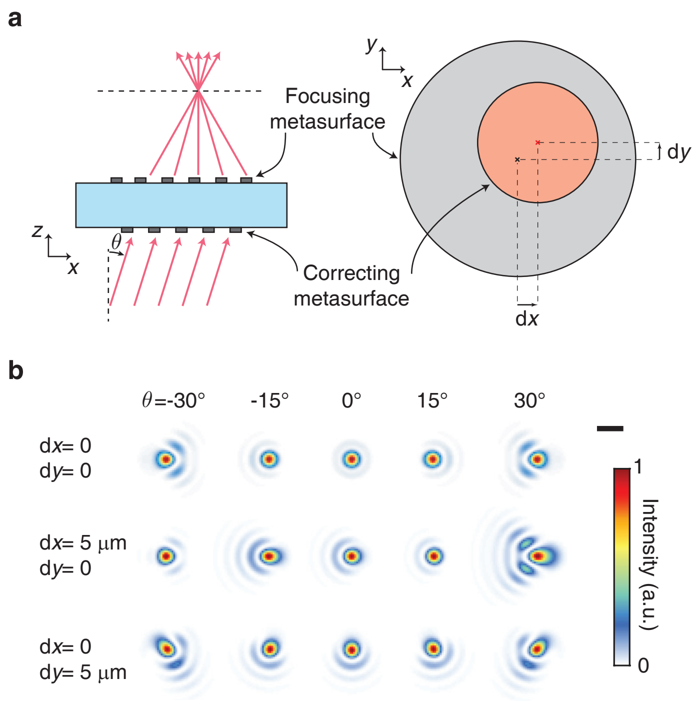


**Supplementary Figure 2**. **Effect of misalignment between the two metasurfaces**. **a**, Schematic illustration of the side and the top views of the metasurface doublet lens. The misalignments along $x$ and $y$ directions (d$x$ and d$y$) are shown in the top view illustration. **b**, Simulated focal plane intensity of the metasurface doublet lens for different misalignments between the metasurfaces and at several different incident angles $\theta$. The aperture and field stops are assumed to be aligned with the metasurfaces on their corresponding sides. Scale bar: 2 µm.

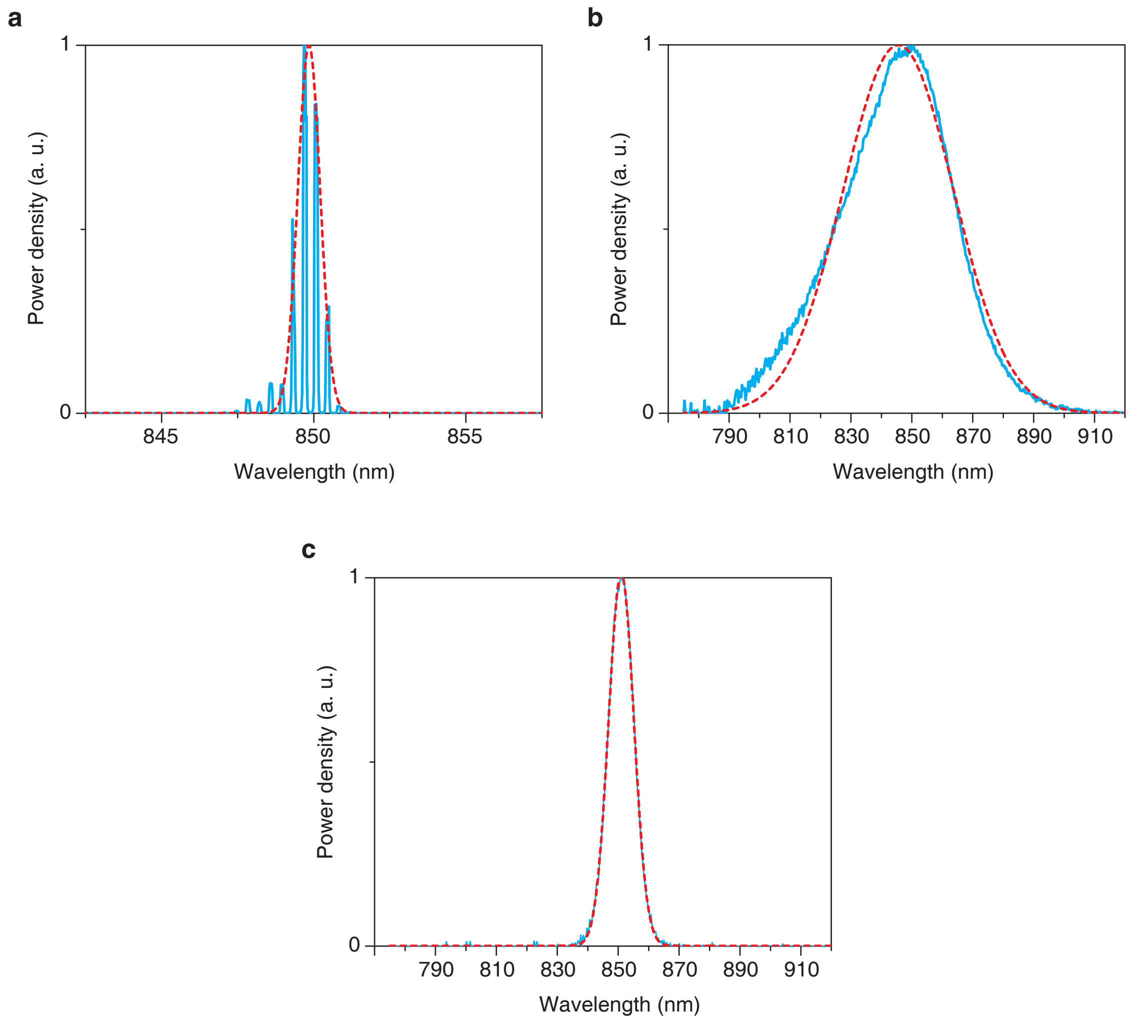


**Supplementary Figure 3**. **Measured spectra of the sources used to characterize the metasurface lenses and the miniature camera**. **a**, Measured spectrum of the laser used in the measurement of incident angle dependent focusing of the metasurface doublet and singlet lenses (Fig. 3a). Different peaks observed in the spectrum correspond to different Fabry-Pérot modes of the laser cavity. **b**, Measured spectrum of the LED used to capture the image shown in Fig. 5c. **c**, Measured spectrum of the filtered LED used as illumination for the images shown in Figs 4b,c,g, and 5d. The solid blue curves show the measured spectra and the dashed red curves represent the best Gaussian function fits. The full width at half maximum (FWHM) bandwidth values for the Gaussian fits are equal to 0.9 nm, 42.7 nm, and 9.8 nm for the spectra shown in **a**, **b**, and **c**, respectively.

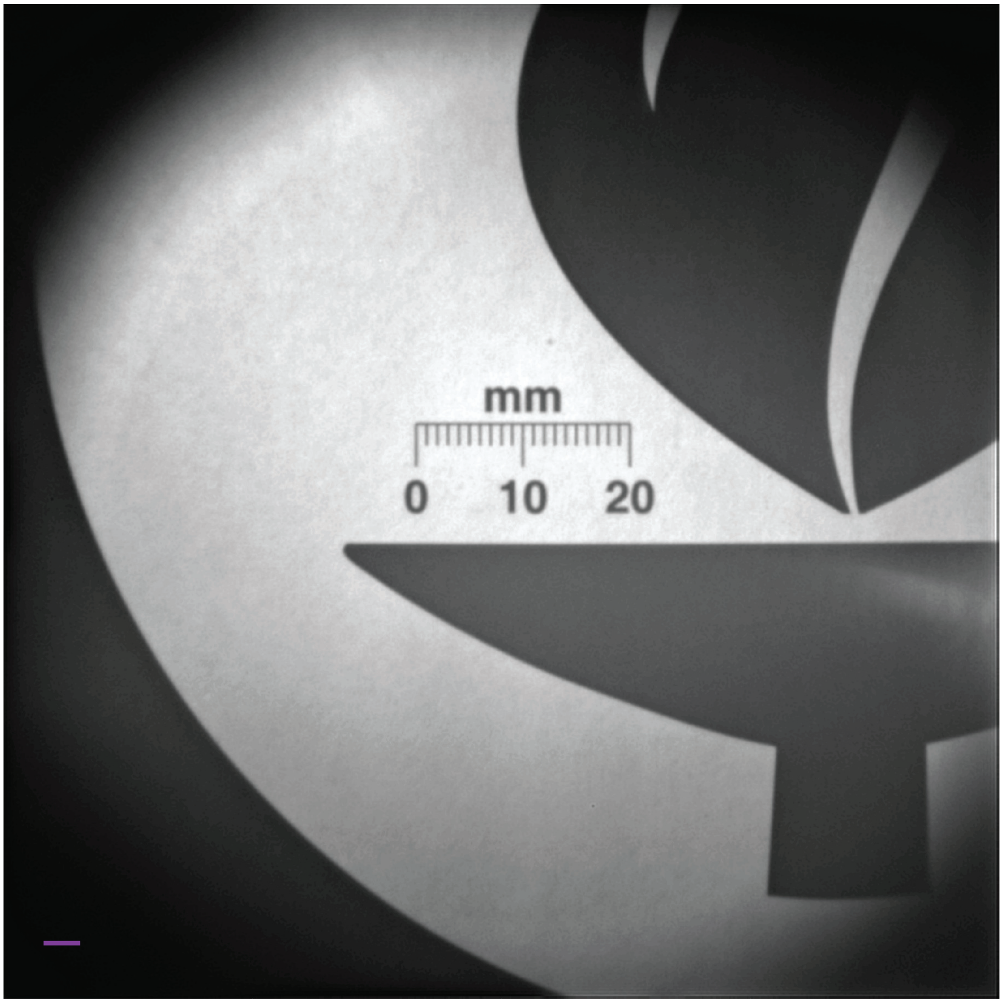


**Supplementary Figure 4**. **Image captured by the metasurface doublet lens.** Image taken using the setup shown in Fig. 4a, but with an objective lens with higher magnification and NA (50×, 0.5 NA). See Methods for the measurement details. The vignetting observed at the corners of the image is due to the limited field of view of the objective lens used to magnify the image. Scale bar: 10 μm.

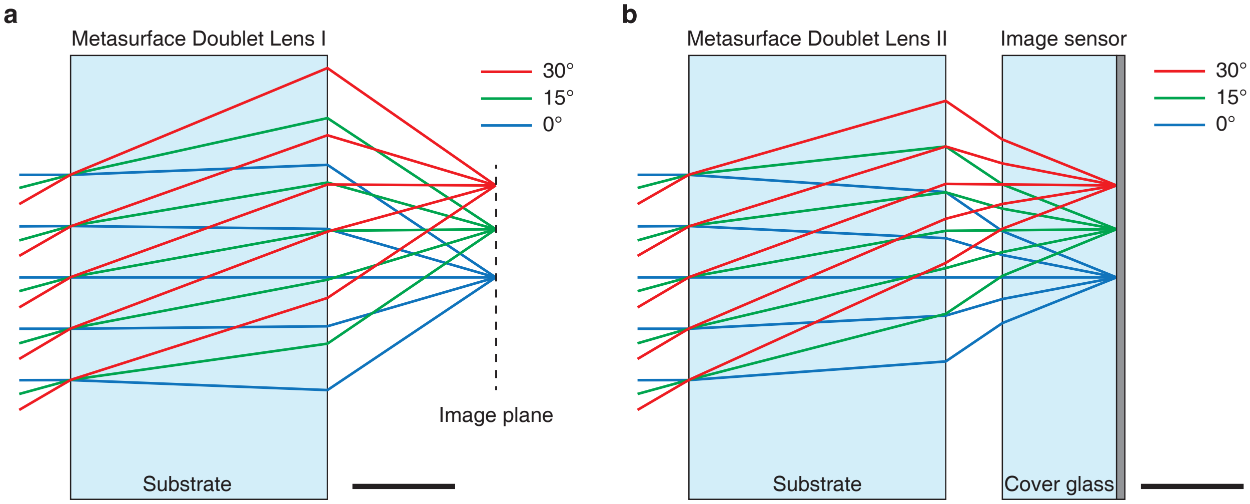


**Supplementary Figure 5**. **Image-space telecentricity of the metasurface doublet lenses. a**, Ray diagram for the metasurface doublet lens I (designed for focusing in air), and **b**, the metasurface doublet lens II (designed for focusing through the cover glass). The correcting and focusing metasurfaces (not shown) are assumed to be patterned on the left and right sides of the substrates, respectively. The chief rays are nearly normal to the image planes (i.e. the lenses are telecentric in the image space), and the angular distributions of the focused rays are independent of the incident angle. Scale bars: 400 μm.

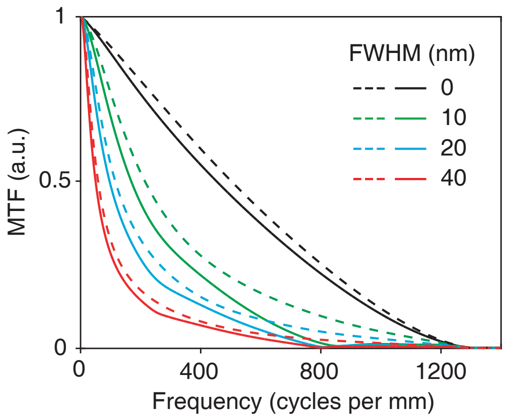


**Supplementary Figure 6**. **Simulated modulation transfer function for the metasurface doublet lens at the incident angle of 15°**. The solid and dashed lines show the modulation transfer function in the tangential plane (along $x$ in Fig. 5a) and sagittal plane (along $y$ in Fig. 5a), respectively.

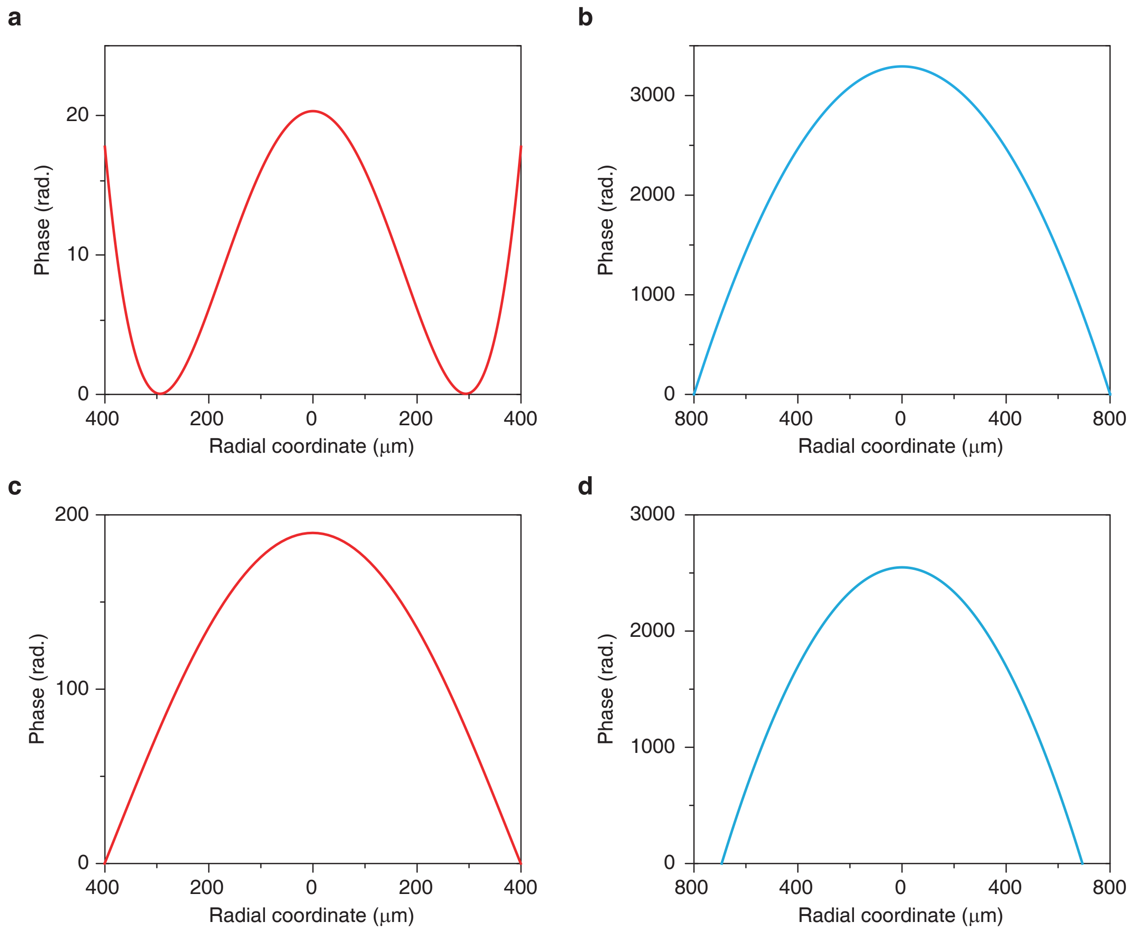


**Supplementary Figure 7**. **Phase profiles of the metasurfaces composing the doublet lenses.** **a**, Phase profile of the correcting and **b**, focusing metasurfaces of the doublet lens I (designed for focusing in air). **c**, and **d**, similar plots as **a** and **b** but for the metasurface doublet lens II which is designed for focusing through the cover glass of a CMOS image sensor (as shown in Figs 4f,g).

## SUPPLEMENTARY TABLES

**Supplementary Table 1 | Phase profile parameters for the metasurface doublet lens I**

| Metasurface | $R$ (μm) | $a_1$ | $a_2$ | $a_3$ | $a_4$ | $a_5$ |
|---|---|---|---|---|---|---|
| Correcting Metasurface | 400 | -71.86 | 57.90 | 9.62 | 1.30 | 0.66 |
| Focusing Metasurface | 800 | -3285.68 | -31.88 | 33.77 | -8.41 | 1.51 |

**Supplementary Table 2 | Phase profile parameters for the metasurface doublet lens II**

| Metasurface | $R$ (μm) | $a_1$ | $a_2$ | $a_3$ | $a_4$ | $a_5$ |
|---|---|---|---|---|---|---|
| Correcting Metasurface | 400 | -225.92 | 31.29 | 3.84 | 0.49 | 0.34 |
| Focusing Metasurface | 700 | -2559.04 | 11.57 | 0.83 | -3.58 | 1.94 |

## SUPPLEMENTARY NOTE 1: IMAGING BANDWIDTH OF METASURFACE LENSES

Here we discuss the relation between the numerical aperture (NA), focal length and bandwidth of a metasurface lens. We consider a metasurface lens with the focal length of $f$ which is designed for operation at the wavelength $\lambda$, and is placed at the distance $f$ from an image sensor. The metasurface lens focuses light with the wavelength of $\lambda + \Delta\lambda$ to the distance of $f - \Delta f$ from the metasurface lens. Because of the phase jumps at the zone boundaries of the metasurface lens, the fractional change in the focal length is equal to the fractional change in the wavelength [1], that is

$$\frac{\Delta f}{f} = \frac{\Delta\lambda}{\lambda}. \tag{1}$$

As $\Delta\lambda$ increases the focal plane of the lens moves further away from the image sensor and the size of the spot recorded by the image sensor increases. As a quantitative measure, we define the bandwidth of the metasurface lens as the wavelength change $\Delta\lambda$ that increases the diameter of the recorded spot by a factor of $\sqrt{2}$ compared to its value at $\lambda$. With this definition, the distance between the image sensor and the focal plane at the wavelength of $\lambda + \Delta\lambda$ is equal to the Rayleigh range $z_0$ of the focused light (i.e. $\Delta f = z_0$). The Rayleigh range is given by

$$z_0 = \frac{\pi w_0^2}{\lambda}, \tag{2}$$

where $w_0$ is the $1/\mathrm{e}^2$ focal spot radius and is inversely proportional to the NA of the metasurface lens

$$w_0 = \frac{\lambda}{2\sqrt{\ln(2)}\mathrm{NA}}. \tag{3}$$

Therefore, the fractional bandwidth of the metasurface lens is given by

$$\frac{\Delta\lambda}{\lambda} = \frac{\pi w_0^2}{\lambda f} = \frac{\pi}{4\ln(2)} \frac{\lambda}{f\mathrm{NA}^2}, \tag{4}$$

and is proportional to $\lambda/(f\mathrm{NA}^2)$.

---

## SUPPLEMENTARY REFERENCES


[1] Arbabi, E., Arbabi, A., Kamali, S. M., Horie, Y. & Faraon, A. Multiwavelength polarization insensitive lenses based on dielectric metasurfaces with meta-molecules. *Optica* **3** (2016).
